# Thoracoscopic and endoscopic cooperative surgery for esophageal gastrointestinal stromal tumor: a case report

**DOI:** 10.1186/s40792-024-02045-y

**Published:** 2024-10-18

**Authors:** Ryo Kanoda, Tomohiro Kikuchi, Akihito Utsumi, Shotaro Mochizuki, Akira Matsuishi, Akinao Kaneta, Azuma Nirei, Hiroyuki Hanayama, Zenichiro Saze, Takuto Hikichi, Yuko Hashimoto, Koji Kono

**Affiliations:** 1 Department of Gastrointestinal Tract Surgery, Fukushima Medical University School of Medicine, 1 Hikariga-Oka, Fukushima City, Fukushima 960-1295 Japan; 2 Department of Endoscopy, Fukushima Medical University Hospital, 1 Hikariga-Oka, Fukushima City, Fukushima Japan; 3 Department of Diagnostic Pathology, Fukushima Medical University School of Medicine, 1 Hikariga-Oka, Fukushima City, Fukushima Japan

**Keywords:** Esophageal gastrointestinal stromal tumor, Thoracoscopic and endoscopic cooperative surgery, TECS

## Abstract

**Background:**

Esophageal gastrointestinal stromal tumors (GISTs) are relatively rare, accounting for 2–5% of all GISTs. Typically, the treatment is surgery in nature. However, no standard procedure established for esophageal GISTs, and in many cases, subtotal esophagectomy or local resection via thoracoscopy or mediastinoscopy is performed. Thoracoscopic and endoscopic cooperative surgery (TECS) is a surgical approach similar to laparoscopic and endoscopic cooperative surgery used for gastric GIST; however, no reports of its use for esophageal GIST have been published to date. We herein report such a case along with a review of past literature.

**Case presentation:**

The patient was a 60-year-old man. Upper gastrointestinal contrast imaging revealed a subepithelial lesion in the esophagus. An 18 × 17 mm subepithelial lesion was identified in the left wall, 35 cm from the upper incisors, during upper gastrointestinal endoscopy, and was diagnosed as a GIST through endoscopic ultrasound-guided fine needle biopsy. TECS was therefore performed. The patient was placed in a prone position with his face to the left. After confirming the lesion under endoscopy and left thoracoscopy, the periesophageal area of the lesion was dissected under thoracoscopy. Subsequently, an endoscopic full-layer resection was performed. Finally, the excision site of the lesion was sutured under thoracoscopy. The operation took a total of 3 h and 22 min, with a blood loss of 50 mL.

**Conclusions:**

The appropriate surgical procedure for esophageal GIST should be considered according to the location and size of the lesion. TECS ensures that the resection margins are secured using an endoscopic or thoracoscopic approach. Furthermore, TECS is minimally invasive, avoiding esophagectomy and reconstruction, which makes it a potential surgical option for esophageal GISTs.

## Background

Esophageal gastrointestinal stromal tumors (GISTs) are a relatively rare disease, accounting for 2–5% of all GISTs [1]. Once a diagnosis of GIST is made, the treatment is thought to be surgical resection in daily practice. However, no standard procedure established for esophageal GISTs, and in many cases, subtotal esophagectomy or local resection via thoracoscopy or mediastinoscopy is performed. In the current report, we present a case of thoracoscopic and endoscopic cooperative surgery (TECS) for esophageal GISTs.

## Case presentation

**Fig. 1:**
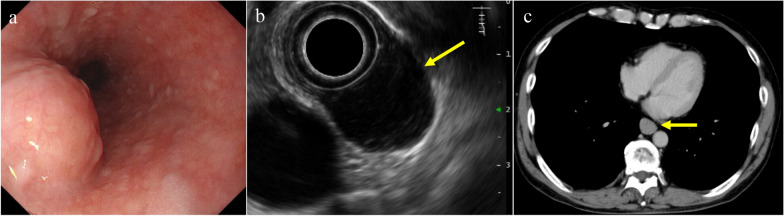
Preoperative findings. **a** Submucosal tumor of 18 mm × 17 mm was observed on the left wall, 35 cm from the upper incisors in the esophagogastroduodenoscopy. **b** Circular tumor continuous with the fourth layer was observed in endoscopic ultrasonography (yellow arrow). **c** 16 mm long nodule was found in the lower esophagus (yellow arrow)

A 60-year-old man was suspected to have a subepithelial lesion (SEL) in the esophagus during a routine upper gastrointestinal contrast study. There were no apparent symptoms. Subsequent esophagogastroduodenoscopy (EGD) performed by his previous physician identified an SEL in the esophageal wall, located 35 cm from the incisors (Fig. 1a). To further investigate this condition, the patient was referred to the Department of Gastroenterology at our hospital. EGD revealed a submucosal tumor measuring 18 mm × 17 mm on the left wall, 35 cm from the upper incisors. Endoscopic ultrasound (EUS) demonstrated that the tumor was located in the fourth layer with poor blood flow (Fig. 1b). EUS-guided fine-needle aspiration (EUS–FNA) was performed. Biopsy specimen showed growth of spindle cells that were immunohistochemically positive for CD117. Biopsy results were positive for CD34, CD117, and vimentin, and negative for AE1/AE3, desmin, SMA, and S100, with a Ki-67 index of 1.5%. A diagnosis of GIST was made, and the patient was referred to our department for surgery.

Contrast-enhanced computed tomography showed a well-defined nodule with a long diameter of approximately 16 mm in the lower esophagus (Fig. 1c). No distant metastases were noted. After consultation with our gastroenterologist and careful consideration of the location, and size of the lesion, as well as surgical impact, we planned to perform TECS following the principles of laparoscopic endoscopic and cooperative surgery for gastric GIST.

**Fig. 2:**
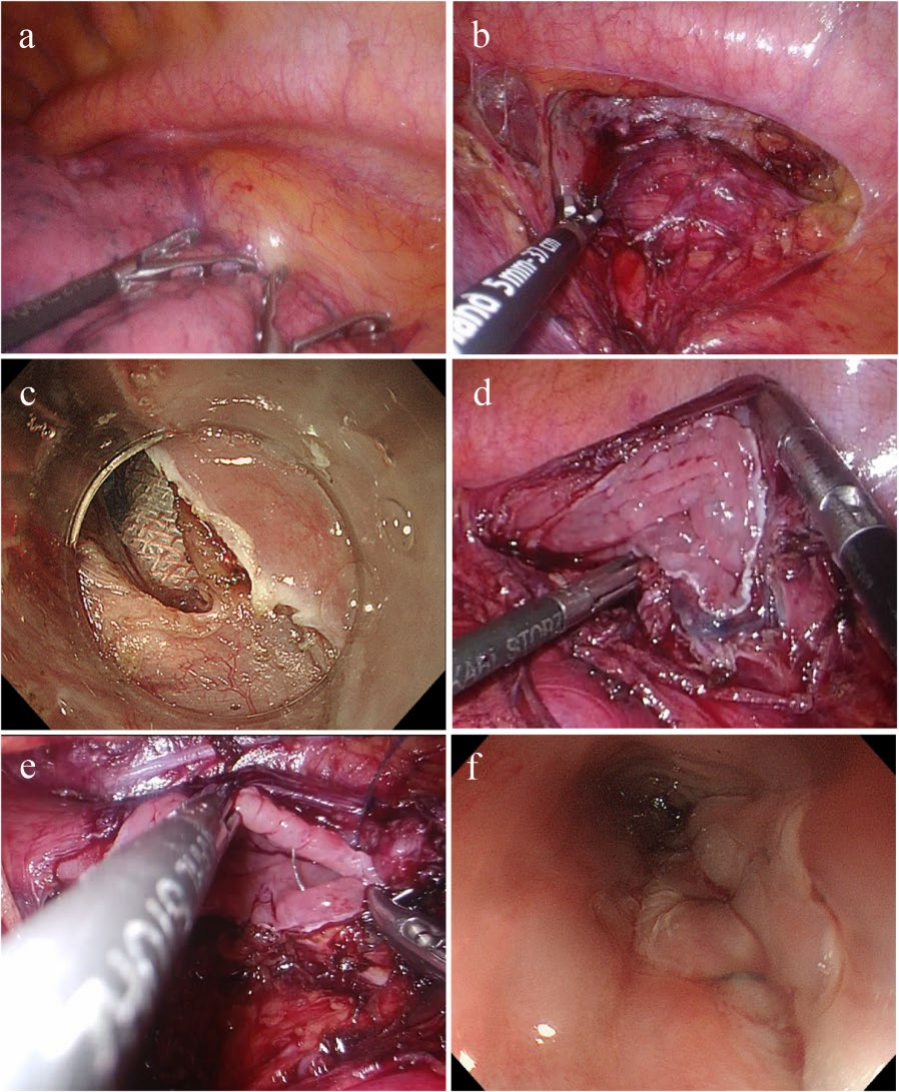
Operative findings. **a** Tumor was not identified. **b** Periesophageal dissection near the tumor. **c** Endoscopic full-layer incision. **d** Defect after resection measured 4.5 cm × 2.5 cm. **e** Sutured in two layers with a barbed suture. **f** Endoscopy after suturing showed slight stenosis, but the scope could be passed through

After induction of general anesthesia, the surgery was started with the patient placed in the prone position, face to the left. To approach the left thoracic cavity, four ports were placed: a 12-mm port in the 5th intercostal space along the left mid-axillary line, a 5-mm port in the 7th intercostal space, another 5-mm port at the anterior axillary line in the 8th intercostal space, and a 12-mm port at the posterior axillary line in the 9th intercostal space. An artificial pneumothorax was initiated at 6 mmHg, and the intrathoracic cavity was examined. No adhesions were found, and the tumor could not be identified macroscopically (Fig. 2a).

When the endoscope was inserted, the tumor was confirmed to be located anteriorly on the left side of the lower esophagus through transillumination. The pleura around the tumor was incised. The surrounding tissues were dissected to expose the esophagus (Fig. 2b). Subsequently, the area around the tumor was marked using an endoscope, and after local administration of a submucosal injection, the mucosa was incised from the oral side. We initiated perforation at a site distant from the heart and commenced a full-thickness incision (Fig. 2c). After performing such an incision for approximately half of the circumference, visualization of the tumor became difficult. Therefore, the remaining half was resected under thoracoscopy. The resected specimen was placed inside plastic bag and removed orally by endoscope.

Minimal resection was conducted; however, the resulting defect measured 4.5 cm along the long axis and 2.5 cm along the short axis (Fig. 2d). We decided to suture the wound along the long axis, considering the difficulty in suturing along the short axis. The closure was performed in a continuous layer-to-layer suture technique with a 4–0 V-Loc™ (Medtronic PLC, MN, USA) (Fig. 2e). The suture site was confirmed endoscopically, and although the lumen had become slightly narrowed, the GIF-H290T scope (Olympus, Tokyo, Japan) could pass through with minimal contact (Fig. 2f). The leak test results were negative. Drains were placed around the anastomotic site and the left lung apex. The procedure took a total of 3 h and 22 min, and had a blood loss of 50 mL.

**Fig. 3:**
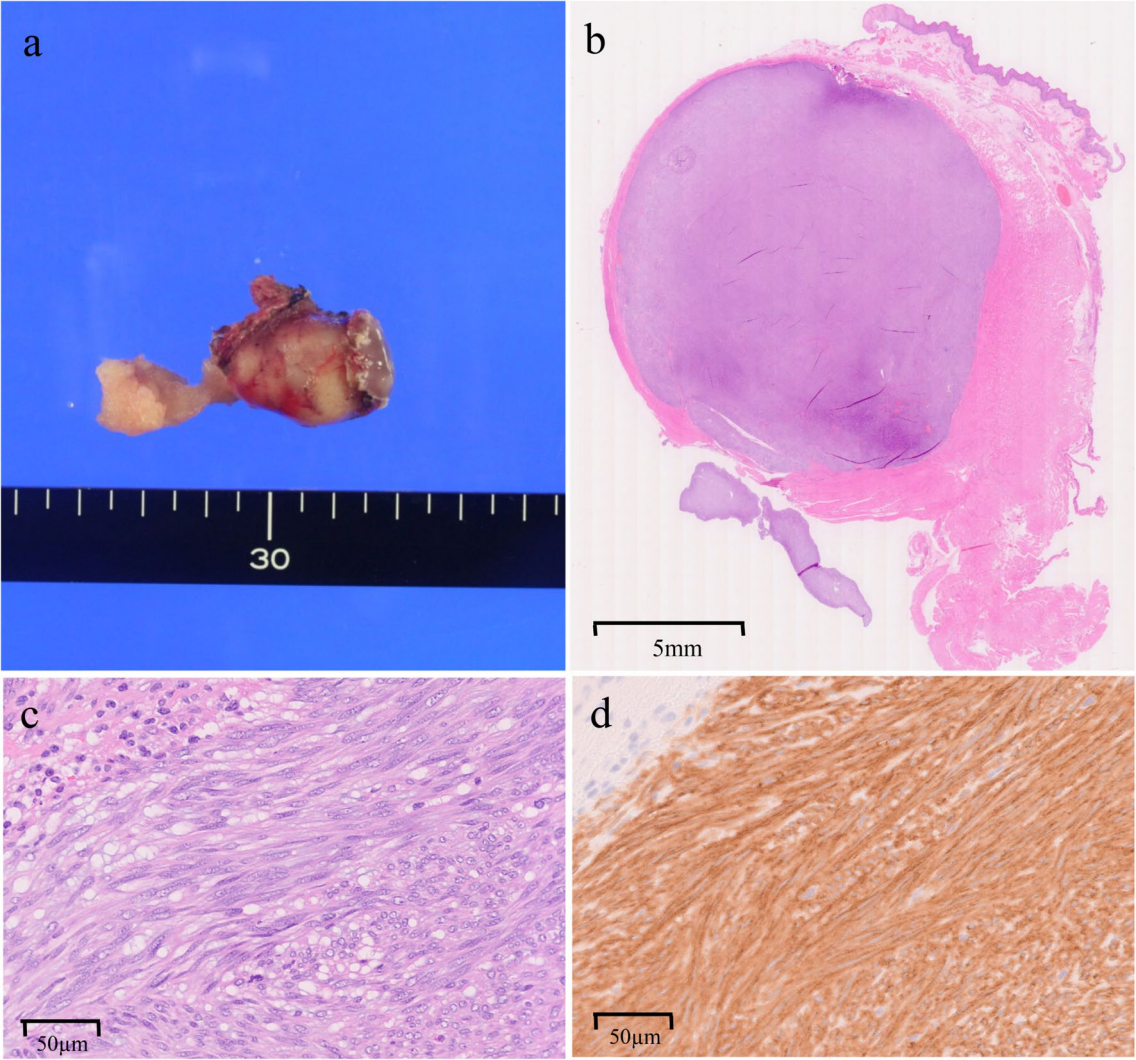
Pathological findings of the resected specimen. **a** Macroscopic image of the resected specimen. The tumor was removed without any damage to the capsule. **b** Findings observed with a loupe after hematoxylin–eosin staining. The tumor had clear borders, and the surgical margins were negative. The size of the tumor was 22 × 14 mm. **c** Hematoxylin and eosin stain shows spindle cells arranged in fascicles. **d** Immunohistochemistry shows tumor-cell positivity for CD117

Macroscopically, the tumor was not exposed on the surgical resection surface in the specimen (Fig. 3a). Pathological findings revealed that the tumor measured 22 × 14 mm. Biopsy specimen showed growth of spindle cells that were immunohistochemically positive for CD117 (Fig. 3b–d). The Ki-67 index was 4%, with two mitoses observed per 50 high-power fields. It was positive for CD34, CD117, and vimentin, and negative for AE1/AE3, desmin, S-100, and SMA, confirming the diagnosis of GIST.

Postoperatively, the patient remained on absolute fasting with the gastric tube in place. On the postoperative day 4 (POD 4), the gastric tube was removed, and oral intake was initiated. On the POD 5, the chest drain was removed, and on the POD 7, an upper gastrointestinal contrast study confirmed the absence of suture insufficiency or significant stenosis. The patient started oral intake on the POD 8, and was discharged on the POD15. EGD performed at follow-up 3 months postoperatively showed no strictures, and the scope passage was unobstructed.

## Discussion

Approximately 70% of esophageal submucosal tumors are leiomyomas and 25% are GISTs [2]. Lott et al. reported that GISTs occur predominantly in males aged < 60 years, and primarily affect the lower esophagus [3]. When diagnosed with a resectable localized GIST without distant metastasis, surgery is the primary treatment. Lymph node dissection is unnecessary, because lymph node metastasis in GIST is rare and does not serve as a prognostic factor. During resection of GIST, it is important to secure resection margins without capsular damage [4].

Currently, no standard procedure is established for esophageal GISTs, and the treatment strategy is determined on a case-by-case basis. In subtotal esophagectomy, securing a definite margin is feasible; however, it entails substantial surgical invasion, posing risks to organ function and potential postoperative quality of life decline. Conversely, local resection is minimally invasive, although concerns may arise about dissemination into the thoracic cavity.

Large tumors may cause large defects in the muscle layer, leading to postoperative esophageal stenosis, dysfunction, and mucosal damage. Notably, successful enucleation using a thoracoscope or robot has been reported in cases of small tumors [5, 6]. However, inward-growing tumors are often challenging to identify from the thoracic cavity, and are unsuitable for enucleation. TECS employs endoscopic and thoracoscopic approaches to ensure resection margins for tumors growing within the lumen and with a small diameter.

In the present case, a tumor, approximately 20 mm in diameter, was found in the lower thoracic esophagus. Since the tumor was discovered incidentally during a medical examination, there were no subjective symptoms. After diagnosing GIST through EUS–FNA, we determined to proceed with surgery. Extensive preoperative discussions with our gastroenterologist led us to conclude that subtotal esophagectomy would be highly invasive. Consequently, we performed TECS after fully explaining the curability and invasiveness to the patient. Preoperative EGD indicated that the tumor was located on the left wall of the esophagus; therefore, we selected a left thoracoscopic approach. During the operation, half of the circumference was resected endoscopically, and the remaining half was resected thoracoscopically. The constant communication with the gastroenterologist enabled us to secure a resection margin and make use of the advantages of this procedure. Because the defect in the longitudinal direction was severe, we sutured the closure along the long axis, and there were no signs of stenosis either symptomatically or endoscopically until postoperative 3 months. For preventing stenosis, suturing along the short axis or at an oblique line would have been preferable; however, this approach was challenging due to the limited space in the mediastinum. According to the modified Fletcher classification, the specimen was categorized as low risk of recurrence. We intend to maintain careful follow-up in the future.

We searched for cases similar to ours in Igaku Chuo Zasshi and PubMed for the period from 2000 to 2024 using the keywords “thoracoscopic and endoscopic cooperative surgery”, we found seven cases [7–10]. Including our own case, eight cases are presented in Table 1. According to the reports, TECS was performed in one case of esophageal schwannoma and leiomyoma and five cases of gastric tube cancer after operation for esophageal cancer, and the resection margins were negative in all cases. All cases of gastric tube cancer were reconstructed through the posterior mediastinal route. The patients were six males and two females aged 49–82 years, with tumors ranging from 13 to 80 mm in diameter, and they were discharged from hospital within 9–25 days, which was relatively early. In one case, thoracotomy was necessary due to severe adhesions. In another case, although TECS was initially considered, the intraoperative endoscope could not be passed through after suturing. Consequently, segmental resection was performed, and the plan was shifted to a two-stage operation. The involvement of the tumor around the circumference was slightly less than half. Although minimal resection was performed, it resulted in stenosis. Regarding the case of leiomyoma, TECS using submucosal tunneling and endoscopic resection was performed for a large tumor with a diameter of 80 mm and involving 2/3 of the circumference. They identified a high risk of stenosis and placed an esophageal stent intraoperatively, which was removed 3 weeks after operation. Patients with large tumors are at a high risk of stenosis, and TECS is not recommended for such a situation. Except for this particular case, TECS was performed in cases with tumor diameters of 3 cm or less. Suturing in the transverse direction is preferred to prevent stenosis in the direction of suturing after excision. However, in some cases, such as the present case, it is necessary to suture in a longitudinal direction, and the circumference of the tumor before surgery is also considered significant.

**Table 1:** Reports of thoracoscopic and endoscopic cooperative surgery for esophageal and gastric tube tumors

Year	First author	Age	Gender	Diagnosis	Tumor size (mm)	Tumor circumference	Procedure	Operative time (min)	Blood loss (mL)	Discharge (POD)
2017	Onodera Y	49	Female	Schwannoma	60	n/a	TECS	498	40	9
2019	Dong T	55	Male	Leiomyoma	80 × 30	2/3	TECS	190	n/a	n/a
2022	Aoyama S	72	Male	Gastric tube cancer	25	1/3	TECS	471	30	25
2022	Aoyama S	68	Male	Gastric tube cancer	18 × 13	1/4	TECS	271	10	14
2022	Aoyama S	72	Female	Gastric tube cancer	30	1/3	TECS transitioned to thoracotomy	470	300	19
2022	Aoyama S	82	Male	Gastric tube cancer	25	More than 1/3 Less than 1/2	TECS divided surgery	n/a	n/a	n/a
2024	Tsuji T	69	Male	Gastric tube cancer	13 × 8	1/3	TECS	216	n/a	12
2024	The present case	60	Male	Esophageal GIST	18 × 17	1/3	TECS	202	50	15

n/a: not available; TECS: thoracoscopic and endoscopic cooperative surgery; POD: post operative day

Among the seven previously reported cases, stenosis was observed during intraoperative endoscopy in two. Preoperative endoscopic findings indicated tumor involvement in 2/3 of the circumference in one case and in 1/3 to half of the circumference 1/3 to half circumferential in the other. When considering the need to secure resection margins, tumors with less than 1/3 circumferential involvement are suitable indications for avoiding stenosis. The surgical margins were negative in all cases. The accumulation of cases is necessary to confirm that TECS does not worsen prognosis compared to other surgical procedures.

## Conclusion

To the best of our knowledge, this is the first report of TECS for esophageal GISTs. It is useful for tumors growing toward the inside of the esophagus, ensuring resection margins without damaging the tumor capsule through endoscopic and thoracoscopic approaches. By avoiding esophageal resection and reconstruction, TECS offers a minimally invasive treatment option and is a reasonable surgical choice for esophageal GISTs. While the indication for TECS remains a matter of debate, we propose that it can be safely performed for tumors measuring 3 cm or less and involving less than 1/3 of the circumference, thereby avoiding stenosis.

## Data Availability

All the data generated or analyzed during this study are included in the article.

## Abbreviations

GIST: Gastrointestinal stromal tumor
TECS: Thoracoscopic and endoscopic cooperative surgery
EGD: Esophagogastroduodenoscopy
EUS–FNA: Endoscopic-ultrasound fine-needle aspiration
n/a: Not available
POD: Postoperative day
